# Recent advances in microbial synthesis of free heme

**DOI:** 10.1007/s00253-023-12968-5

**Published:** 2024-01-09

**Authors:** Shaomei Yang, Zihao Guo, Jiuyu Sun, Jingxuan Wei, Qinyuan Ma, Xiuzhen Gao

**Affiliations:** School of Life Sciences and Medicine, Shandong University of Technology, 266 Xincun West Road, Zibo, 255000 China

**Keywords:** Heme, Biosynthetic pathway, Fermentation production, *Bacillus subtilis*, Secretion

## Abstract

**Abstract:**

Heme is an iron-containing porphyrin compound widely used in the fields of healthcare, food, and medicine. Compared to animal blood extraction, it is more advantageous to develop a microbial cell factory to produce heme. However, heme biosynthesis in microorganisms is tightly regulated, and its accumulation is highly cytotoxic. The current review describes the biosynthetic pathway of free heme, its fermentation production using different engineered bacteria constructed by metabolic engineering, and strategies for further improving heme synthesis. Heme synthetic pathway in *Bacillus subtilis* was modified utilizing genome-editing technology, resulting in significantly improved heme synthesis and secretion abilities. This technique avoided the use of multiple antibiotics and enhanced the genetic stability of strain. Hence, engineered *B. subtilis* could be an attractive cell factory for heme production. Further studies should be performed to enhance the expression of heme synthetic module and optimize the expression of heme exporter and fermentation processes, such as iron supply.

**Key points:**

• *Strengthening the heme biosynthetic pathway can significantly increase heme production.*

• *Heme exporter overexpression helps to promote heme secretion, thereby further promoting excessive heme synthesis.*

• *Engineered B. subtilis is an attractive alternative for heme production.*

## Introduction

Heme is an iron-containing porphyrin compound that is essential for the survival of virtually all living systems (Gallio et al. 2021). It serves as a prosthetic group in proteins involved in multiple biological processes, such as oxygen transport and storage, electron transfer, and oxidative stress detoxification (Beas et al. 2022). Heme has extensive application value. In the field of healthcare, since heme iron is a better bioavailable and tolerable form of iron, it can be suitable for supplementation in pregnancy for the treatment of anemia (Abbas et al. 2020). In the field of food coloring, heme is a safe and natural pigment that is used to replace carcinogenic chromogen nitrite and synthetic pigments. Moreover, with the development of plant-based meat alternatives, heme-containing proteins can be used to generate meat flavors and/or aromas in a variety of food products during the cooking process (Ahmada et al. 2022). In the field of pharmaceuticals, heme is a raw material used for semisynthesis of hematoporphyrin and its derivatives, as well as for protoporphyrin sodium, which is used for treatment of diseases (Cannon 1993; Yarra et al. 2019).

**Fig. 1:**
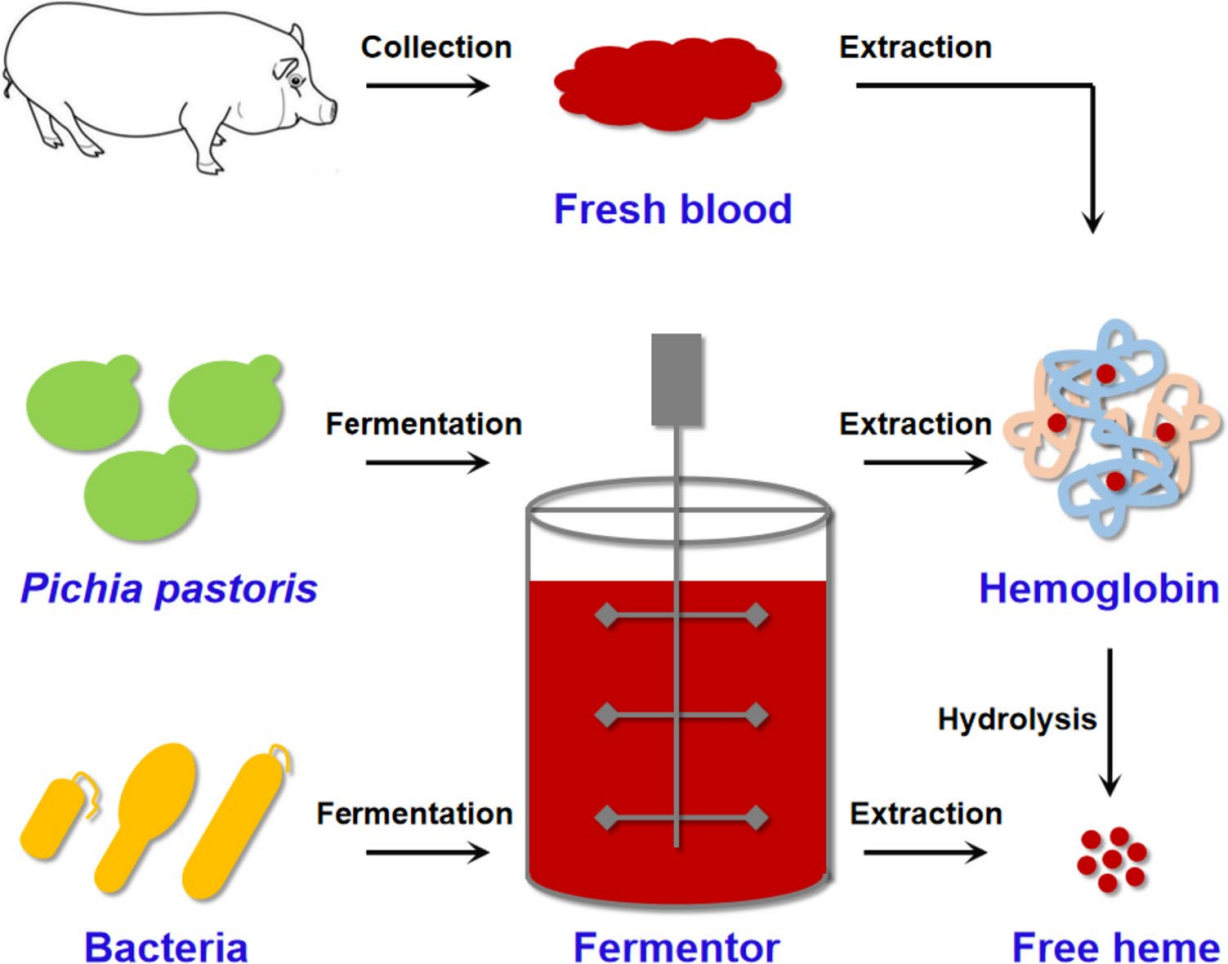
The main free heme production methods

The global heme market size is expected to reach $530 million by 2026. The traditional heme production method extracts hemoglobin from the blood of pigs or other animals and then prepares free heme through enzymatic hydrolysis. However, collection, transportation, and storage of animal blood are relatively troublesome, and obtaining hemoglobin from blood by a solid-phase extraction method is expensive, low-yielding, and time-consuming (Jia et al. 2017). Therefore, developing microbial cell factories to produce heme is more advantageous. For example, Impossible Foods Incorporation (Redwood City, CA) has developed a *Pichia pastoris* expression system to realize the industrial-scale production of soy leghemoglobin (Fraser et al. 2018). Shao et al. have achieved a leghemoglobin titer of 3.5 g/L by enhancing globin expression and heme biosynthesis (Shao et al. 2022). Zhao et al. summarized the current strategies for microbial synthesis of hemoglobin, such as promoting heme synthesis, discovering superior hemoglobins, balancing heme synthesis and globins expression (Zhao et al. 2021). However, hemoglobin is mainly used for producing artificial meat products and needs to be hydrolyzed to obtain free heme. In recent years, more and more researchers have focused on developing bacterial cell factories through synthetic biology to directly produce free heme (Fig. 1). The free heme production of engineered *Escherichia coli* was 1,034 mg/L (Choi et al. 2022), the highest yield of heme produced by microbial fermentation reported so far.

The current review provides a concise and comprehensive summary of the progress of microbial synthesis of heme, including the biosynthetic pathway of free heme and its fermentation production with different engineered bacteria, i.e., *E. coli*, *Corynebacterium glutamicum*, and *Bacillus subtilis*. In microorganisms, heme biosynthesis is tightly regulated, showing highly cytotoxic effect at accumulation > 1 µM (Nishinaga et al. 2021; Sassa 2004). Heme is mainly accumulated inside the cell during flask fermentation and outside the cell during fed-batch fermentation in the fermenter (Ko et al. 2021; Yang et al. 2023; Zhao et al. 2018). The secretion ability of heme can be improved by increasing the expression of heme exporter. Based on the comparative investigations of the characteristics of these three engineered bacteria, it is proposed that *B. subtilis* is expected to be an efficient cell factory for heme production. In addition, strategies for further improving microbial synthesis of heme are proposed, such as enhancing the expression of heme synthetic module, finding the endogenous heme exporter or dynamically regulating the expression of the heterologous heme exporter, and optimizing the fermentation processes such as iron supply.

## Biosynthetic pathway of free heme

**Fig. 2:**
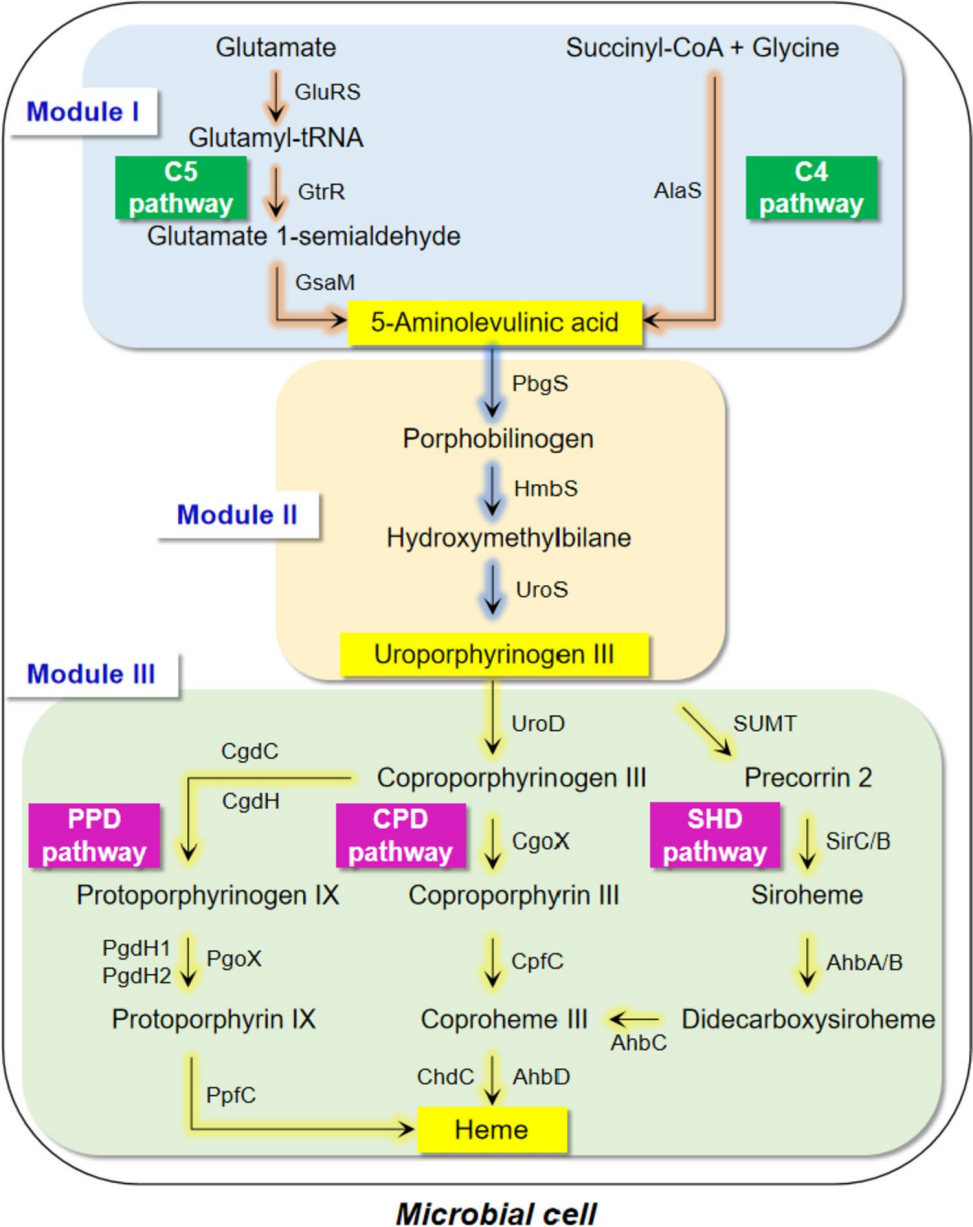
Biosynthetic pathway of heme in microorganisms. GluRS, glutamyl-tRNA synthase; GtrR, glutamyl-tRNA reductase; GsaM, glutamate-1-semialdehyde-2,1-aminomutase; AlaS, 5-aminolevulinic acid synthase; PbgS, porphobilinogen synthase; HmbS, hydroxymethylbilane synthase; UroS, uroporphyrinogen (urogen) III synthase; UroD, urogen III decarboxylase; CgoX, coproporphyrinogen (coprogen) oxidase; CpfC, coproporphyrin ferrochelatase; ChdC, coproheme decarboxylase; CgdC, oxygen-dependent coprogen III oxidase; CgdH, oxygen-independent coprogen III dehydrogenase; PgoX, protoporphyrinogen oxidase; PgdH1 and PgdH2, protoporphyrinogen dehydrogenase; PpfC, protoporphyrin ferrochelatase; SUMT, S-adenosyl-L-methionine urogen III methyltransferase; SicC, precorrin-2 dehydrogenase; SirB, sirohydrochlorin ferrochelatase; AhbA and AhbB, siroheme decarboxylase; AhbC, Fe-coproporphyrin synthase; AhbD, coproheme decarboxylase

The biosynthetic pathway of heme is divided into three modules in microorganisms, including 5-aminolevulinic acid (ALA), uroporphyrinogen (urogen) III, and heme synthetic modules (Fig. 2).

### ALA synthetic module

ALA is an important precursor for heme synthesis with two biosynthetic pathways of C4 and C5 (Fig. 2). The C4 pathway exists in humans, animals, fungi, and purple non-sulfur phototrophic bacteria (Stojanovski et al. 2019; Zhao et al. 2021), such as *Rhodobacter capsulatus* and *Rhodobacter sphaeroides*. ALA synthase (AlaS) is the only enzyme in this pathway that catalyzes the condensation of glycine and succinyl-CoA to form ALA. The C5 pathway is present in archaea, plants, and other bacteria (Layer 2021; Zhao et al. 2021), such as *Salmonella arizona*, *E. coli*, and *B. subtilis*. It involves glutamyl-tRNA synthase (GluRS), glutamyl-tRNA reductase (GtrR), and glutamate-1-semialdehyde 2,1-aminomutase (GsaM), catalyzing L-glutamate to generate ALA. In *S. arizona* and *E. coli*, HemA is sensitive to hydrolase and is regulated by heme. GtrR^M^ is obtained by inserting two lysine residues (KK) between the Thr2 and Leu3 residues of native GtrR, relieving feedback inhibition (Yu et al. 2015; Zhao et al. 2018). In *B. subtilis*, GtrR is negatively regulated by the negative regulatory protein HemX, decreasing the steady-state cellular concentration of GtrR protein by controlling its synthesis rate (Johansson and Hederstedt 1999; Schroder et al. 1994). This regulation is released by knocking out *hemX* (Yang et al. 2023). In addition, there are a few microorganisms with both of the C4 and C5 pathways, such as *Euglena gracilis* (Weinstein and Beale 1983).

### Urogen III synthetic module

The synthesis of ALA to urogen III includes three enzymatic reactions and is a common pathway among all microorganisms. First, two ALA molecules are condensed and cyclized to generate one porphobilinogen molecule catalyzed by porphobilinogen synthase (PbgS) (Hansson et al. 1991; Lu et al. 2010). Then, four porphobilinogen molecules react with one water molecule to form one hydroxymethylbilane molecule and release four ammonia molecules catalyzed by hydroxymethylbilane synthase (HmbS) (Hansson et al. 1991). Finally, urogen III synthase (UroS) catalyzes the dehydration of hydroxymethylbilane to generate urogen III (Hansson et al. 1991; Stamford et al. 1995). That is, eight ALA molecules synthesize one urogen III molecule. In *E. coli* and *B. subtilis*, PbgS, HmbS, and UroS are encoded by *hemB*, *hemC*, and *hemD*, respectively. Zhao et al. have constructed the expression plasmid pRSF-*hemB*-*hemC*-*hemD* to promote the conversion of ALA to urogen III, thereby promoting the synthesis of heme in *E. coli* (Zhao et al. 2018). Yang et al. have promoted heme synthesis by integrating *hemC*-*hemD*-*hemB* into the genome of *B. subtilis* (Yang et al. 2023).

### Heme synthetic module

Depending on the species, there are three synthetic pathways from urogen III to heme, including protoporphyrin-dependent (PPD), coproporphyrin-dependent (CPD), and siroheme-dependent (SHD) pathways (Fig. 2) (Dailey et al. 2017; Layer 2021). The PPD pathway exists in both Gram-negative and Gram-positive bacteria, while the CPD pathway only exists in some Gram-positive bacteria, such as *C. glutamicum* and *B. subtilis*. The SHD pathway is considered the most ancient route for heme synthesis as it is present in archaea, sulfate-reducing bacteria. At present, there are no research reports on the heme synthesis through modifying the SHD pathway.

Urogen III decarboxylase (UroD) is a common enzyme in the PPD and CPD pathways, catalyzing the removal of four CO_2_ molecules from urogen III to generate coproporphyrinogen (coprogen) III. In the PPD pathway, coprogen III is oxidized to protoporphyrinogen IX by oxygen-dependent coprogen III decarboxylase (CgdC) or oxygen-independent coprogen III dehydrogenase (CgdH). Then, protoporphyrinogen IX is oxidized to form protoporphyrin IX, catalyzed by protoporphyrinogen oxidase (PgoX) or protoporphyrinogen dehydrogenase (PgdH1/PgdH2). Ultimately, protoporphyrin ferrochelatase (PpfC) catalyzes the insertion of Fe^2+^ into protoporphyrin IX to produce heme. In *E. coli*, genes *hemE*, *hemF*, *hemG*, and *hemH* encode UroD, CgdC, PgdH1, and PpfC, respectively. Zhao et al. (2018) constructed the plasmid pET-*hemE*-*hemF*-*hemG*-*hemH* to increase the expression levels of four endogenous enzymes, i.e., UroD, CgdC, PgdH1, and PpfC, thereby promoting the conversion of urogen III to heme in *E. coli*. In the CPD pathway, coprogen III reacts with three O_2_ molecules to form coproporphyrin III catalyzed by coprogen III oxidase (CgoX). In *B. subtilis*, CgoX can also oxidize protoporphyrinogen IX to generate protoporphyrin IX, with lower oxidization rate than that of oxidization of coprogen III to form coproporphyrin III (Corrigall et al. 1998; Hansson and Hederstedt 1994). Then, coproporphyrin ferrochelatase (CpfC) catalyzes the insertion of Fe^2+^ into coproporphyrin III to produce Fe-coproheme III. Finally, coproheme is decarboxylated to heme by coproheme decarboxylase (ChdC). In *C. glutamicum*, UroD, CgoX, CpfC, and ChdC are encoded by *hemE*, *hemY*, *hemH*, and *hemQ*, respectively. Ko et al. have reinforced the endogenous CPD pathway by overexpressing *hemE*, *hemY*, *hemH*, and *hemQ* (pX2-*hemA*^M^-*hemL-alaS-dtxR-hemE* and pMTC-*hemY*-*hemH*-*hemQ*-*hrtBA*) to promote the synthesis of urogen III to heme in *C. glutamicum* (Ko et al. 2021).

In microorganisms, excessive accumulation of heme (> 1 µM) (Sassa 2004) is highly cytotoxic due to its Lewis acidity, redox activity, and hydrophobicity (Nishinaga et al. 2021), causing deleterious effects, such as membrane disorder, oxidative stress damage, lipid oxidation, and DNA damage (Choby and Skaar 2016). To maintain the intracellular homeostasis of heme, heme oxygenase catalyzes the degradation of heme to produce bilirubin (Park et al. 2014; Shibahara 1988).

## Fermentation production of free heme with engineered bacteria

To increase the production of heme, many researchers have engineered a synthetic heme pathway. Table 1 shows an overview of previous reports on heme production by engineered microbial strains.

**Table 1:** Overview of previous reports on heme production using engineered microbial strains

Microorganism	Genetic modification	Gene source	Culture condition	Total heme titer	Heme secretion ratio	References
*E. coli*	pACmod-*hemA*-*hemB*-*hemC*-*hemD*, pBBR-*hemE-hemF*, pUCmod-*hemH*	*hemA* (AlaS) from *Rhodobacter capsulatus*; *hemB* (PbgS), *hemC* (HmbS), *hemD* (UroS), and *hemF* (CgdC) from *E. coli*; *hemE* (UroD) from *Synechocystis sp.*; *hemH* (CpfC) from *B. subtilis*	Flask fermentation	3.3 ± 0.3 μmol/L	ND	(Kwon et al. 2003)
	pTrc-*hemA*-*coaA*	*hemA* (AlaS) from *Rhodobacter sphaeroides*; *coaA* (pantothenate kinase) from* E. coli*	Flask fermentation	9.1 μmoL/g DCW	ND	(Pranawidjaja et al. 2015)
	pETDuet-*hox1*-*pcyA*, pET-28a-*hemB*, pCDFDuet-*hemG*-*hemH*	*hox1* (hemeoxygenase) and *pcyA* (phycocyanobilin: ferredoxinoxidoreductase) from *Synechocystis sp.*; *hemB* (PbgS), *hemG* (PgdH1), and *hemH* (PpfC) from* E. coli*	Flask fermentation	10.30 ± 0.39 μmol/L	ND	(Ge et al. 2018)
	pCDF-*hemA*^M^-*hemL*, pRSF-*hemB*-*hemC*-*hemD*, pET-*hemE*-*hemF*-*hemG*-*hemH*, pACYC-*ccmABC*, Δ*ldhA*, Δ*pta*, Δ*yfeX*	*hemA*^M^ (GtrR^M^), *hemL* (GsaM), *hemB* (PbgS), *hemC* (HmbS), *hemD* (UroS), *hemE* (UroD), *hemF* (CgdC), *hemG* (PgdH1), *hemH* (PpfC), and *ccmABC* (heme exporter) from* E. coli*	6.6 L Fed-batch fermentation	239.2 ± 7.2 mg/L	63.3%	(Zhao et al. 2018)
				1034.3 mg/L	45.5%	(Choi et al. 2022)
*C. glutamicum*	pECXK99E-*hemA*^M^-*hemL-gltX*	*hemA*^M^ (GtrR^M^) from *Salmonella arizona*; *hemL* (GsaM) from *E. coli*; *gltX* (GluRS) from *C. glutamicum*	Flask fermentation	4.22 ± 0.62 mg/L	ND	(Yu et al. 2015)
	pSL360-*hemA*	*hemA* (AlaS) from *R. sphaeroides*	Flask fermentation	1.2 ± 0.2 μmoL/g DCW	ND	(Choi et al. 2017)
	pEKEx2-*hemA*^M^-*hemL-dtxR*	*hemA*^M^ (GtrR^M^) from *Salmonella typhimurium*; *hemL* (GsaM) from *E. coli*; *dtxR* (transcriptional regulator diphtheria toxin repressor) from *C. glutamicum*	Flask fermentation	29.57 ± 2.46 mg/L	ND	(Ko et al. 2018)
	pX2-*hemA*^M^-*hemL-alaS-dtxR-hemE*, pMTC-*hemY*-*hemH*-*hemQ*-*hrtBA*, Δ*hrrS*, Δ*htaA*, Δ*hmuT*	*hemA*^M^ (GtrR^M^) from *Salmonella typhimurium*; *hemL* (GsaM) from *E. coli*; *alaS* from *R. capsulatus*; *dtxR* (transcriptional regulator diphtheria toxin repressor), *hemE* (UroD), *hemY* (CgoX), *hemH* (CpfC), *hemQ* (ChdC), and *hrtBA* (heme transporter) from *C. glutamicum*	2 L Fed-batch fermentation	111.87 ± 6.48 mg/L	91.25%	(Ko et al. 2021)
				129.81 ± 10.18 mg/L	73.01%	
				309.18 ± 16.43 mg/L	78.58%	
*B. subtilis*	Δ*hemX*, Δ*ywfM*::P_*43*_-*hemA*, Δ*rocG*::P_*43*_-*alaS*, Δ*gcvTP*::P_*lapS*_-*hemC*-*hemD*-*hemB*, Δ*ywjI*::P_*lapS*_-*ccmABC*, Δ*nasF*, Δ*hmoA*, Δ*hmoB*	*hemA* (GtrR), *hemC* (HmbS), *hemD* (UroS), and *hemB* (PbgS) from *B. subtilis*; *alaS* (AlaS) from *Bradyrhizobium japonicum*; *ccmABC* (heme exporter) from* E. coli*	2 L Fed-batch fermentation	150.78 ± 0.59 mg/L	92%	(Yang et al. 2023)
				87.77 ± 0.32 mg/L	88%	
			10 L Fed-batch fermentation	248.26 ± 6.97 mg/L	89%	

ND Not detected

### Fermentation by engineered *E. coli*

Among numerous microbial cell factories, *E. coli* is the most popular and user-friendly workhorse primarily due to its genetic amenability and well-developed bioprocessing strategies. The native biosynthetic pathway of heme in *E. coli* includes the C5, urogen III, and PPD pathways. Kwon et al. were the first to have used *E. coli* as the chassis and constructed three expression plasmids to overexpress AlaS from *R. capsulatus*, endogenous PbgS, HmbS, UroS (pACmod-*hemA*-*hemB*-*hemC*-*hemD*), and UroD from *Synechocystis sp.*, and endogenous CgdC (pBBR-*hemE*-*hemF*), and CpfC from *B. subtilis* (pUCmod-*hemH*). By introducing the heterogeneous C4 pathway and strengthening the urogen III and PPD pathways, the heme yield of the final engineered strain was 3.3 ± 0.3 μmol/L after 48 h of fermentation (Kwon et al. 2003). Pranawidjaja et al. have constructed plasmid pTrc-*hemA-coaA* to co-express AlaS from *R. sphaeroides* and endogenous pantothenate kinase CoaA in *E. coli* W3110, where heme production reached 9.1 μmol/g dry cell weight (DCW) (Pranawidjaja et al. 2015). Ge et al. have constructed three plasmids to overexpress ferredoxin-dependent hemeoxygenase gene *hox1*, phycocyanobilin:ferredoxinoxidoreductase gene *pcyA* from *Synechocystis sp.*, endogenous *hemB*, *hemG*, and *hemH* (pETDuet-*hox1-pcyA*, pET-28a-*hemB*, and pCDFDuet-*hemG-hemH*). The constructed *E. coli* S5 strain produced 10.30 ± 0.39 μmol/L heme (Ge et al. 2018).

Zhao et al. have constructed four expression plasmids (pCDF-*hemA*-*hemL*, pRSF-*hemB*-*hemC*-*hemD*, pET-*hemE*-*hemF*-*hemG*-*hemH*, and pACYC-*ccmABC*) to strengthen the endogenous C5, urogen III, and PPD pathways and overexpress heme exporter CcmABC. Furthermore, phosphate acetyl transferase gene *pta*, lactate dehydrogenase gene *ldhA*, and putative heme-degrading enzyme gene *yfeX* were also knocked out. The cells were harvested by centrifugation with the supernatant colected to detect the production of extracellular free heme. The cell pellet was resuspended into 1 M NaOH, and the cell suspension was ultrasonicated for 10 min in an ice bath and centrifuged again; the supernatant of the cell lysate was collected to examine the production of intracellular free heme. The amount of heme was quantified using high performance liquid chromatography (HPLC) equipped with a reverse-phase C18 column and a UV-detector set at 400 nm. The mobile phase was 30% methanol with 0.1% trifluoroacetic acid and a gradient elution was performed at 0.4 mL/min (Zhao et al. 2018). The engineered *E. coli* produced 239.2 ± 7.2 mg/L of total heme with 151.4 ± 5.6 mg/L of extracellular heme during fed-batch fermentation in a 6.6-L fermenter (Zhao et al. 2018). Furthermore, its heme production reached up to 1,034 mg/L by increasing cell density, regular iron supplementation, and supply of excess feeding solution (Choi et al. 2022). However, the release of endotoxin from the cell walls of *E. coli* precipitates an acute inflammatory response that often leads to shock and death (Epstein et al. 1999; Kilbourn et al. 1993). Thus, heme produced by the engineered *E. coli* needs to be purified to remove endotoxin prior to its use in the healthcare, food, and pharmaceutical industries.

### Fermentation by engineered *C. glutamicum*

*Corynebacterium glutamicum* is a Gram-positive actinobacterium with the advantages of stable fermentation performance and no endotoxins. It is generally recognized as a safe (GRAS) microorganism, which has become a model organism in industrial biotechnology due to its use in large-scale amino acid production (Lei et al. 2021; Ogata and Hirasawa 2021). The native biosynthetic heme pathways in *C. glutamicum* include the C5, urogen III, PPD and CPD pathways. Yu et al. were the first to have used *C. glutamicum* as the chassis, with the heme yield of the constructed recombinant strain of 4.22 ± 0.62 mg/L via co-expression of *hemA*^M^ (GtrR^M^) from *S. arizona*, *hemL* (GsaM) from *E. coli*, and endogenous *gltX* (GluRS) (Yu et al. 2015). Choi et al. have expressed *hemA* (AlaS) from *R. sphaeroides* alone (pSL360-*hemA*) in *C. glutamicum*, with the heme yield of 1.2 ± 0.2 μmoL/g DCW (Choi et al. 2017). Ko et al. have constructed *C. glutamicum* CGPB07 strain by co-overexpressing *hemA*^M^ (GtrR^M^) from *S. typhimurium*, *hemL* (GsaM) from *E. coli*, and endogenous *dtxR* encoding the transcriptional regulator diphtheria toxin repressor. As a result, heme concentration reached 29.57 ± 2.46 mg/L (Ko et al. 2018).

Subsequently, Ko et al. have used a two-plasmid expression system to overexpress the C5 pathway (*hemA*^M^ derived from *Salmonella typhimurium*, and *hemL* derived from *E. coli*), the heterologous C4 pathway (*alaS* derived from *R. capsulatus*), the diphtheria toxin repressor protein DtxR, the endogenous CPD pathway (*hemE*, *hemY*, *hemH*, and *hemQ*) and the heme transporter HrtBA, and knocked out heme-binding protein genes (*hrrS*, *htaA*, and *hmuT*). The *C. glutamicum* recombinant strain (pX2-*hemA*^M^-*hemL-alaS-dtxR-hemE*, pMTC-*hemY*-*hemH*-*hemQ*-*hrtBA*, Δ*hrrS*, Δ*htaA*, and Δ*hmuT*) resulted in 309.18 ± 16.43 mg/L of total heme with 242.95 ± 11.45 mg/L of extracellular heme in 2-L fed-batch fermentation using modified CGXII medium supplemented with ethambutol for changing the lipid component (Ko et al. 2021). Ethambutol is a first-line drug for treating tuberculosis (Blumberg et al. 2003) that has been reported to cause Leber's hereditary optic neuropathy in patients carrying mitochondrial DNA mutations (Kim et al. 2010). Hence, the purification process of heme produced by engineered *C. glutamicum* should be performed prior to its application in the food industry (Ko et al. 2021).

### Fermentation by engineered *B. subtilis*

*Bacillus subtilis* is an attractive alternative due to its status as a GRAS strain commonly used for efficiently synthesizing various high-value added compounds, such as pyrimidine nucleosides (Fan et al. 2018; Zhu et al. 2015) and vitamins (Liao et al. 2021; Yang et al. 2019). It is widely used in food, feed, medicine, agriculture, and industry (Su et al. 2020; Xiang et al. 2020). In *B. subtilis*, the native biosynthetic pathway of heme also includes the C5, urogen III, PPD, and CPD pathways, where the CPD pathway is the main pathway for synthesizing heme (Dailey et al. 2017).

**Fig. 3:**
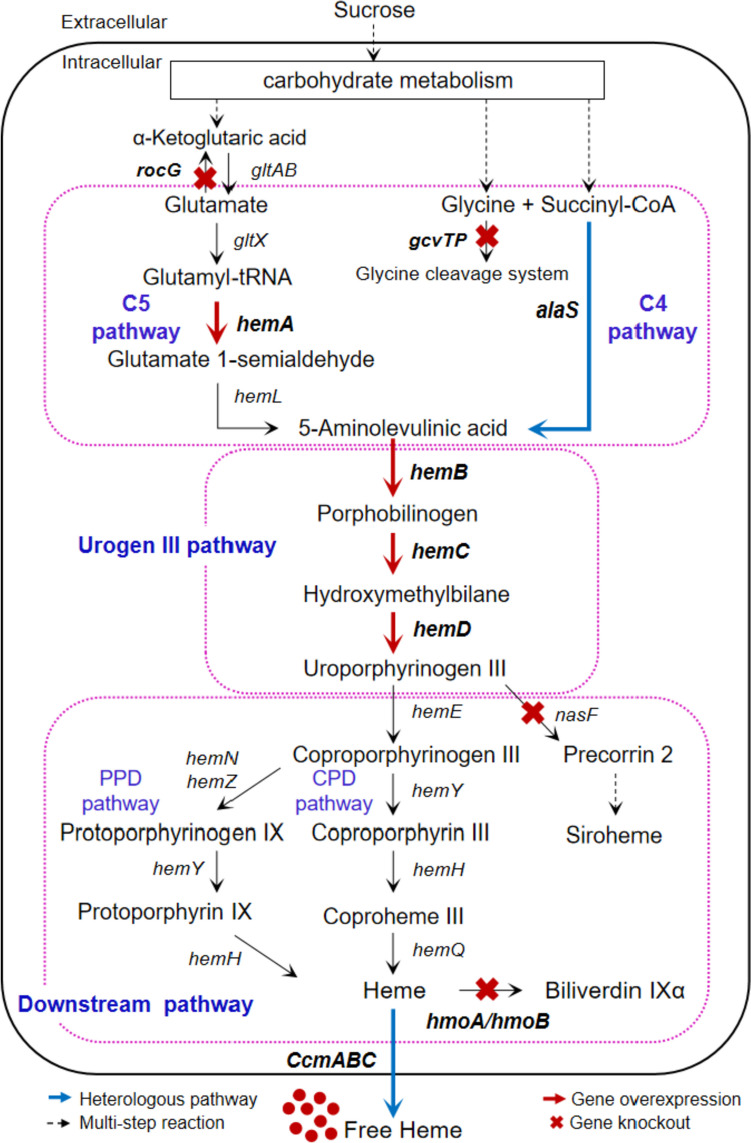
Schematic diagram of heme biosynthetic pathway and overall engineering strategy in *B. subtilis* (Yang et al. 2023). *rocG*, encoding glutamate dehydrogenase; *gltAB*, encoding glutamate synthase; *gltX*, encoding glutamyl-tRNA synthase; *hemA*, encoding glutamyl-tRNA reductase; *hemL*, encoding glutamate-1-semialdehyde-2,1-aminomutase; *gcvTP*, encoding aminomethyltransferase (glycine cleavage system protein T) and glycine decarboxylase; *alaS*, 5-aminolevulinic acid synthase; *hemB*, encoding porphobilinogen synthase; *hemC*, encoding hydroxymethylbilane synthase; *hemD*, encoding urogen III synthase; *hemE*, encoding urogen III decarboxylase; *hemY*, encoding coprogen/protoporphyrinogen oxidase; *hemH*, encoding coproporphyrin/protoporphyrin ferrochelatase; *hemQ*, encoding coproheme decarboxylase; *hemN* and *hemZ*, encoding oxygen-independent coprogen III dehydrogenase; *nasF*, encoding uroporphyrinogen methyltransferase; *hmoA* and *hmoB*, encoding heme monooxygenase; *ccmABC*, encoding heme exporter from *E. coli*

Yang et al. were the first to have used *B. subtilis* as the chassis and engineered its heme synthetic pathway through genome modification (Fig. 3). Strengthening the endogenous C5 pathway by sequentially knocking out the negative regulatory protein gene *hemX*, overexpressing the endogenous glutamyl-tRNA reductase gene *hemA*, and knocking out the glutamate dehydrogenase gene *rocG*, increased the heme production by 427%. However, introducing the heterologous C4 pathway by expressing four different heterologous AlaSs had no significant effect on the heme synthesis. It is possible that ALA synthesized by increasing the metabolic flux of the endogenous C5 pathway was sufficient for heme synthesis, and the metabolic flux of urogen III pathway was low, which limited the positive effect of the C4 pathway on heme synthesis. Strengthening the urogen III pathway was reinforced by overexpressing endogenous *hemC* (encoding HmbS)*-hemD* (encoding UroS)*-hemB* (encoding PbgS), while the heme yield was increased by 39%. In addition, methyltransferase gene *nasF* was knocked out to block the competitive consumption of urogen III, while monooxygenase hemes *hmoA* and *hmoB* were knocked out to block the intracellular degradation of heme, where heme production was increased by 11%, 26%, and 11%, respectively. The final recombinant strain BSH11 secreted 221.83 ± 4.71 mg/L of heme, which was 89% of total heme (248.26 ± 6.97 mg/L) produced during fed-batch fermentation in a 10-L fermenter (Yang et al. 2023). Notably, although the CPD pathway in strain BSH11 was not further modified, its extracellular heme production was comparable to that of *C. glutamicum*, which secreted the highest level of free heme (242.95 ± 11.45 mg/L) (Ko et al. 2021). In addition, *B. subtilis* strains constructed via genome editing (Liu et al. 2008) instead of using multi-plasmid expression systems (Ko et al. 2021; Zhao et al. 2018) avoided the addition of multiple antibiotics, simplifying the heme purification process and improving the genetic stability of engineered strains (Bu et al. 2021), demonstrating the advantages of *B. subtilis* in efficient heme production on a commercial scale. Although its yield was not high enough, using *B. subtilis* to produce heme from a food safety perspective is still worthwhile to explore.

## Strategies for further improving microbial synthesis of free heme

Heme synthetic module is crucial for the heme synthesis, usually by strengthening overexpression of the PPD or CPD pathway to promote its biosynthesis. However, the efficient synthesis of heme might lead to excessive accumulation of heme in the cell, which would produce cytotoxicity, thus inhibiting the strain growth, which is counterproductive for heme synthesis. Therefore, heme secretion is a major drawback limiting its production by microbial fermentation. In addition, amplifying and optimizing the fermentation process can also improve microbial synthesis of heme.

### Enhancement of heme synthetic module

In *E. coli*, the heme synthetic module only includes the PPD pathway, consisting of reactions catalyzed by UroD (*hemE*), CgdC (*hemF*), PgdH1 (*hemG*), and PpfC (*hemH*). Zhao et al. have investigated the impact of individual expression and co-expression of these four genes with different combination sequences on heme synthesis, and demonstrated that the expression of pET-*hemE*-*hemF*-*hemG*-*hemH* promoted heme synthesis (Zhao et al. 2018). In *C. glutamicum*, the heme synthetic module includes the PPD and CPD pathways, the latter consisting of reactions catalyzed by UroD (*hemE*), CgoX (*hemY*), CpfC (*hemH*), and ChdC (*hemQ*). Ko et al. overexpressed *hemY*, *hemH*, *hemQ*, *hemH-hemQ*, and *hemY-hemH-hemQ*, respectively, and found that both heme production and specific growth rate were decreased (Ko et al. 2021). However, engineered *C. glutamicum* they constructed still overexpressed four enzymes of the CPD pathway to promote heme synthesis. In *B. subtilis*, the CPD pathway consisting of reactions catalyzed by UroD, CgoX, CpfC, and ChdC is the main pathway for heme synthesis, which has not been modified in the engineered *B. subtilis* (Yang et al. 2023). They have only made engineering modifications to the ALA synthetic module and urogen III synthetic module. Therefore, if the metabolic flux of CPD pathway is further increased, it may significantly improve the heme synthesis ability of *B. subtilis*.

### Overexpression of heme exporter

In *E. coli*, CcmABCDEFGH is responsible for the synthesis of cytochrome *c*, in which CcmABC has the function of heme transfer (Kranz et al. 2009; Sutherland et al. 2021). Zhao et al. have constructed plasmid pACYC-*ccmABC* to induce the expression of CcmABC and transferred it into recombinant *E. coli* HAEM6 to obtain strain HAEM7. Intracellular and extracellular heme titers of HAEM7 were 6.8 ± 0.2 mg/L and 1.4 ± 0.1 mg/L after 48 h of flask fermentation, respectively, which were slightly higher than those of HAEM6 (6.6 ± 0.2 mg/L and 1.3 ± 0.2 mg/L). However, its total heme titer reached 239.2 ± 7.2 mg/L, comprising 87.7 ± 1.7 mg/L of intracellular heme and 151.4 ± 5.6 mg/L (63.3%) of extracellular heme during the fed-batch fermentation (Zhao et al. 2018).

In *C. glutamicum*, the HrtBA protein is responsible for transporting heme from the inside to the outside. Ko et al. have constructed plasmid pMTC-*hemY*-*hemH*-*hemQ*-*hrtBA* to induce the expression of native HrtBA and transferred it into the recombinant *C. glutamicum* ALSdt-YHQ to obtain strain ALSdt-YHQBA. It produced 2.96 ± 0.22 mg/L of extracellular heme and 32.00 ± 0.37 mg/L of total heme, 2.78- and 1.12-fold increases compared to those of ALSdt-YHQ. The final recombinant strain ΔSAT:ALSdtE-YHQBA produced 111.87 ± 6.48 mg/L of total heme in ethambutol-treated fed-batch fermentation, including 102.08 ± 6.28 mg/L (91.25%) of extracellular heme (Ko et al. 2021).

In *B. subtilis*, the gene encoding heme exporter remains unknown. We integrated the *ccmABC* genes from *E. coli* under the control of constitutive promoter P_*lapS*_ into the genome of *B. subtilis* BSH7, resulting in a 27% decrease in heme production. However, total heme titer of strain BSH11, which we constructed on the basis of BSH8, reached 248.26 ± 6.97 mg/L during fed-batch fermentation with 221.83 ± 4.71 mg/L (89%) of extracellular heme (Yang et al. 2023). Perhaps finding an endogenous heme exporter or dynamically regulating the expression of a heterologous heme exporter will be more effective in promoting the synthesis and secretion of heme in *B. subtilis*.

### Optimization of fermentation processes

In the final step of the PPD pathway, PpfC catalyzes the binding of protoporphyrin IX with iron ions to form heme (Dailey et al. 2017). In the third step of the CPD pathway, CpfC catalyzes the binding of coproporphyrin III with iron ions to generate Fe-coproheme III (Dailey et al. 2017). Therefore, it indicates that iron is an important component of heme. Pranawidjaja et al. (2015) performed the batch culture of *E. coli* W3110 harboring pTrc-*hemA*-*coaA* with or without the addition of ferrous ion, and found that the addition of ferrous ion enabled the strain to produce 5.8% more heme than that of control. In addition, iron is also the cofactor of many enzymes involved in the respiration, tricarboxylic acid (TCA) cycle, and DNA biosynthesis (Andrews et al. 2003). However, excessive ferrous ions not only causes metabolic disorders, but also catalyzes the decomposition of hydrogen peroxide to generate the highly reactive hydroxyl radical, resulting in oxidative damage and ultimate cell death (Grass 2006). Choi et al. (2022) conducted fed-batch fermentation using strain *E. coli* HAEM7 with FeSO_4_∙7H_2_O of 0.5 g/L, 1 g/L, 2 g/L, 4 g/L, and 8 g/L in the feed solution, respectively, to achieve the total heme production of 201.8 mg/L, 362.6 mg/L, 330.9 mg/L, 353.9 mg/L, and 317.4 mg/L, respectively. Therefore, optimizing the supplementation concentration of ferrous ions is crucial for improving heme synthesis. Furthermore, in both *E. coli* and *B. subtilis*, the iron acquisition and storage is controlled by the ferric uptake regulator (Fur) for maintaining iron homeostasis (Baez et al. 2022; Steingard et al. 2023). Alternatively, the improved expression of Fur could further enhance the heme synthesis.

In addition, the production and secretion ratio of heme change when the composition of fermentation medium is optimized. For example, heme titer of engineered *E. coli* HAEM7 up to 1,034 mg/L, with a secretion ratio of 45.5%, by replacing carbon source glucose with glycerol, optimization of iron concentration in culture medium and feed solution, and optimization of the pH control, etc. (Choi et al. 2022). The engineered *C. glutamicum* ΔSAT:ALSdtE-YHQBA was used to conduct fed-batch fermentation in the modified CGXII (mCGXII) medium, heme titer was 177.79 ± 14.20 mg/L, including 129.81 ± 10.18 mg/L of secreted heme (73.01%) (Ko et al. 2021). However, its heme titer was 111.87 ± 6.48 mg/L and secretion ratio was 91.25% in mCGXII medium supplemented with 10 mg/L ethambutol, with a maximum titer of 309.18 ± 16.43 mg/L, including 242.95 ± 11.45 mg/L of secreted heme (78.58%) in mCGXII-Tr (mCGXII + 10 g/L tryptone) medium (Ko et al. 2021). When the engineered *B. subtilis* strain BSH11 was used to conduct fed-batch fermentation in a 2-L fermenter, the heme yield was 150.78 ± 0.59 mg/L and the secretion ratio was 92% (Yang et al. 2023). However, when sucrose was replaced with glucose of the same concentration, the heme yield was 87.77 ± 0.32 mg/L, with the secretion ratio of 88% (Yang et al. 2023). When the fed-batch fermentation was carried out in a 10-L fermenter, glucose served as the carbon source in the early and middle stages of fermentation, and was replaced with sucrose in the later stage. The maximum heme yield reached 248.26 ± 6.97 mg/L, and its secretion ratio was 89% (Yang et al. 2023). Since only the fermentation carbon source was optimized so far, optimizing fermentation parameters, such as iron supply and composition of feeding medium, will further improve heme synthesis in *B. subtilis*.

## Conclusions and perspectives

Heme has important physiological functions and many application values. The production of free heme by microbial fermentation is more advantageous and attractive than animal blood extraction. Currently, engineered *E. coli* constructed using four expression plasmids result in the highest production of free heme, accompanied by endotoxin secretion. Heme production by engineered *C. glutamicum* constructed using two expression plasmids is the second best method, where ethambutol needs to be added during fermentation. Hence, the produced heme is not suitable for use in the food industry. Instead of using multi-plasmid expression systems, engineered *B. subtilis* constructed via genome editing avoids the addition of antibiotics and inducers during fermentation and enhances the genetic stability of the strain. The produced heme can thus be used in the food industry, although its yield is relatively low.

Generally, excessive heme synthesis is achieved by strengthening its biosynthetic pathway, and heme synthetic module of engineered *B. subtilis* has not been further modified. In the microbial synthesis of heme, improving the secretion of heme and optimizing fermentation processes for scale-up fermentation are the key to further increasing heme production. Inducing the expression of endogenous heme exporter in *E. coli* and *C. glutamicum* can promote heme secretion and synthesis. Although the constitutive expression of *E. coli*'s heme exporter in *B. subtilis* can also promote heme secretion, finding endogenous heme exporter or dynamically regulating the expression of heterologous heme exporter will further improve heme secretion and synthesis. In addition, optimizing the carbon nitrogen ratio of fed medium, DO, and other fermentation parameters to achieve high-density fermentation will greatly improve heme synthesis. In summary, engineered *B. subtilis* is expected to become an attractive cell factory for the production of free heme on a commercial scale.

## Data Availability

Not applicable.
